# *In Vitro* Identification of Novel Plasminogen-Binding Receptors of the Pathogen *Leptospira interrogans*

**DOI:** 10.1371/journal.pone.0011259

**Published:** 2010-06-22

**Authors:** Monica L. Vieira, Marina V. Atzingen, Tatiane R. Oliveira, Rosane Oliveira, Daniel M. Andrade, Silvio A. Vasconcellos, Ana L. T. O. Nascimento

**Affiliations:** 1 Centro de Biotecnologia, Instituto Butantan, São Paulo, São Paulo, Brazil; 2 Interunidades em Biotecnologia, Instituto de Ciências Biomédicas, Universidade de São Paulo, São Paulo, São Paulo, Brazil; 3 Laboratorio de Zoonoses Bacterianas do VPS, Faculdade de Medicina Veterinária e Zootecnia, Universidade de São Paulo, São Paulo, São Paulo, Brazil; Max Planck Institute for Infection Biology, Germany

## Abstract

**Background:**

Leptospirosis is a multisystem disease caused by pathogenic strains of the genus *Leptospira*. We have reported that *Leptospira* are able to bind plasminogen (PLG), to generate active plasmin in the presence of activator, and to degrade purified extracellular matrix fibronectin.

**Methodology/Principal Findings:**

We have now cloned, expressed and purified 14 leptospiral recombinant proteins. The proteins were confirmed to be surface exposed by immunofluorescence microscopy and were evaluated for their ability to bind plasminogen (PLG). We identified eight as PLG-binding proteins, including the major outer membrane protein LipL32, the previously published rLIC12730, rLIC10494, Lp29, Lp49, LipL40 and MPL36, and one novel leptospiral protein, rLIC12238. Bound PLG could be converted to plasmin by the addition of urokinase-type PLG activator (uPA), showing specific proteolytic activity, as assessed by its reaction with the chromogenic plasmin substrate, D-Val-Leu-Lys 4-nitroanilide dihydrochloride. The addition of the lysine analog 6-aminocaproic acid (ACA) inhibited the protein-PLG interaction, thus strongly suggesting the involvement of lysine residues in plasminogen binding. The binding of leptospiral surface proteins to PLG was specific, dose-dependent and saturable. PLG and collagen type IV competed with LipL32 protein for the same binding site, whereas separate binding sites were observed for plasma fibronectin.

**Conclusions/Significance:**

PLG-binding/activation through the proteins/receptors on the surface of *Leptospira* could help the bacteria to specifically overcome tissue barriers, facilitating its spread throughout the host.

## Introduction

Leptospirosis is an emerging infectious disease with worldwide distribution and a zoonosis that is of human and veterinary concern. Pathogenic *Leptospira* are the etiological agents of leptospirosis, a disease with greater incidence in tropical and subtropical regions [1], [2], [3], [4], [5]. Leptospires cause chronic infection in domestic and wild mammalian species that harbor the bacteria in their renal tubules, shedding them through the urine into the environment, thus constituting a source of re-infection in other animals. Humans are accidental and terminal hosts in the transmission process of leptospirosis [3], [6]. Leptospirosis represents a great economic burden as it affects the public health system and livestock [3].

The molecular pathogenesis of leptospirosis is poorly understood. To date, few virulence factors contributing to the pathogenesis of the disease have been identified [7], [8], [9]. It is well documented that the interaction of pathogens with the extracellular matrix (ECM) may play a primary role in the colonization of host tissues, as long-lasting infections may occur if microorganisms reach the sub-epithelial tissues [10]. Recently, the ability of the leptospires to adhere to ECM macromolecules has been shown, and some adhesins, ECM-binding proteins, have been identified [9], [11], [12], [13], [14], [15], [16], [17], [18], [19]. However, under normal conditions, ECM is not exposed to bacteria. The proteolytic activity achieved by subversion of host proteases by pathogens, such as plasmin, has been demonstrated to be important in various bacterial infections [20]. Plasmin is a broad-spectrum serine protease component of the fibrinolytic system, which has the zymogen plasminogen (PLG) as its main component.

In our previous work, we found that *Leptospira* species are able to bind PLG and generate plasmin, in the presence of activator, on the outer surface *in vitro* [21], an activity already shown for the pathogenic spirochetes *Borrelia* and *Treponema* [22], [23], [24], [25], [26]. We have also revealed that leptospires express multiple PLG-binding proteins, as reported for other microorganisms. Recently, Verma et al. [27] have shown that the protein LenA of *L. interrogans* [14], formerly LfhA/Lsa24 [12], [27], is a surface receptor for human plasminogen.

In the present study, we further evaluated the ability of 14 recombinant proteins of *Leptospira* to mediate binding to PLG *in vitro*. Eight of the 14 assayed were identified as novel PLG-receptor proteins, including the major outer membrane protein LipL32 [28], the previously published rLIC12730, rLIC10494, Lp29, Lp49, LipL40 and MPL36 [29], [30], [31], and one novel leptospiral protein rLIC12238. Bound PLG could be converted to plasmin by the addition of urokinase-type PLG activator (uPA), showing specific proteolytic activity. We also show that PLG interaction with LipL32, also an ECM adhesin, was affected by collagen type IV but not plasma fibronectin. Our data provide an array of novel leptospiral receptors for human plasminogen, which constitutes an important step for the understanding of the molecular pathogenesis and infection process of these bacteria.

## Results

### Selection of putative surface proteins from genome sequences

The rationale for protein selection was mostly based on cellular localization, since surface proteins are potential receptors for PLG. Fourteen proteins available in our laboratory were selected: twelve were previously published, LipL32 [28], [32], LipL40, MPL36 [29], rLIC10509 [33], Lp29, Lp49 [30], Lsa27 [11], MPL21, MPL17 [31], rLIC10494, rLIC12730 [12], and Lsa63 [19], and two are novel proteins, rLIC12922 and rLIC12238. Table 1 summarizes the features of the selected proteins and gene conservation within the sequenced genomes [34], [35], [36], [37].

**Table 1:** Gene locus, protein name, NCBI reference sequence, features, gene conservation, sequence of the primers employed for DNA amplification, and molecular mass of expressed recombinant proteins.

Gene locus^1^	Recombinant protein given name	NCBI reference sequence number^2^	Description/Function	Conservation (identity)^3^	Sequence of primers for PCR amplification	Recombinant protein molecular mass [70]
LIC11352	LipL32^a^	YP_001316	Major outer membrane protein (MOMP), LipL32 lipoprotein	Lai (100%); LBH (98%)	F: 5′ CACCGGTGCTTTCGGTGGTCTG 3′	30.2
					R: 5′ ATTACTTAGTCGCGTCAGAAGC 3′	
LIC10314	Lsa63^b^	YP_000304	Conserved hypothetical protein with Borrelia_P83 domain	Lai (98%); LBH (87%)	F: 5′ GGATCCTTATTTTCTCAGGAAAG 3′ (BamH I)	63.0
				LBP (39%)	R: 5′ GGTACCCTAAGGTTTAATTTTTTT 3′ (Kpn I)	
LIC10509	rLIC10509^c^	YP_000493	Putative lipoprotein	Lai (98%)	F: 5′ CCGGGATCCAAAAAGAGCAAAGAAG 3′ (BamH I)	22.0
					R: 5′ GGTACCCTACTCGAGACAGCCAGGACCTTC 3′ (Kpn I)	
LIC12892	Lp29^d^	YP_002808	Putative lipoprotein		F: 5′ CTCGAGGCAGTACATTACAATCTTGCT 3′ (Xho I)	29.6
					R: 5′ CCATGGCTCTTAGGAGCCTGGAAA 3′ (Nco I)	
LIC10793	Lp49^d^	YP_000772	Putative lipoprotein, Surface antigen	Lai (99%); LBH (86%)	F: 5′ CTCGAGAGCGGAGACTTTTCTTTACTT 3′ (Xho I)	49.1
					R: 5′ CCATGGTTAAAAACCATCTCTACGATAAAC 3′ (Nco I)	
LIC12895	Lsa27^e^	YP_002811	Putative lipoprotein	Lai (79%)	F: 5′ GGATCCCTGAAATATACGAA 3′ (EcoRI)	27.0
					R: 5′ GAATTCTTACTGTTCTCCTTC 3′ (BamHI)	
LIC13131	MPL21^a^^,^ f	YP_003039	Hypothetical protein with Ycel domain	Lai (98%); LBP (45%)	F: 5′ CACCACGTCTCAAAGTTACGCTTCAG 3′	21.9
					R: 5′ TTCTCACCATCCAGCTCGG 3′	
LIC10765	MPL17^a^^,^ f	YP_000745	Conserved hypothetical protein	Lai (100%); LBH (80%)	F: 5′ CACCGAAAGTCCCGTAAGGTTCAAA 3′	15.4
				LBP (41%)	R: 5′ TGCAGGAGTTCCCACATTTTA 3′	
LIC10091	LipL40^a^	YP_000088	Putative lipoprotein	Lai (100%); LBH (84%)	F: 5′ CCATGGGACTCGAGACGCCTCCTCCTAAAGATCC 3′	39.0
					R: 5′ CTCCATGGTCATTTCAAAACTTCTACGGGGC 3′	
LIC10054	MPL36^a^	YP_000054	Putative lipoprotein with Rare lipoprotein A (RplA) like domain	Lai (100%); LBH (88%)	F: 5′ CACCACGTCTTGTGCGTCGGTAGAG 3′	35.1
				LBP (50%)	R: 5′ CCAAGTATTCTATTTATACGTCCGAG 3′	
LIC10494	rLIC10494^g^	YP_000478	Putative lipoprotein	Lai (99%)	F: 5′ CACCACTGCTAGGGCTGCAGAAA 3′	25.8
					R: 5′ ACTTTGAGAGCTTCGTCTCGT 3′	
LIC12730	rLIC12730^g^	YP_002650	Hypothetical protein with TPR domain and 4 NHL repetition	Lai (100%); LBH (90%)	F: 5′ CACCAGTTCTGACGGACTTCCCAA 3′	77.4
				LBP (37%)	R: 5′ TCTTGCGAATGAGTTGATCC 3′	
LIC12922	rLIC12922^h^	YP_002837	Conserved hypothetical protein	Lai (100%); LBH (89%)	F: 5′ CACCGAATCACTCAACAGAGTCATTGC 3′	40.0
				LBP (48%)	R: 5′ ATCAATCTAAATGAAACGTCTCTTC 3′	
LIC12238	rLIC12238^h^	YP_002173	Putative lipoprotein	Lai (100%); LBH (81%)	F: 5′ CTCGAGTGTTTTAAACCTACCGGAG 3′ (Xho I)	17.6
				LBP (39%)	R: 5′ AAGCTTCTACTTCATCGCTTTTTCTATATC 3′ (Hind III)	

1 http://aeg.lbi.ic.unicamp.br/world/lic/ [35].

2 http://www.ncbi.nlm.nih.gov/protein/.

3 Protein BLAST - http://www.ncbi.nlm.nih.gov/blast/Blast.cgi [71], [72].

a Previously published by Gamberini et al. [29].

b Vieira et al. [19].

c Previously published by Gómez et al. [33].

d Previously published by Neves et al. [30].

e Previously published by Longhi et al. [11].

f Previously published by Oliveira et al. [31].

g Previously published by Barbosa et al. [12].

h This work; Lai: *L. interrogans* serovar Lai [34]; LBH: *L. borgpetersenii* serovar Hardjo-bovis [36]; LBP: *L. biflexa* serovar Patoc [37].

### Expression and purification of recombinant proteins

**Figure 1:**
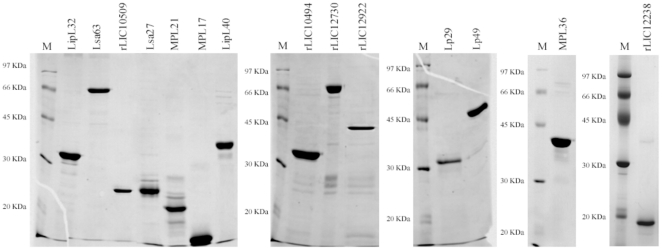
SDS-PAGE analysis of purified recombinant proteins. Recombinant proteins were expressed in *E. coli* BL21 (DE3) or SI and purified from the bacterial cell lysate using Ni^2+^-charged chelating Sepharose columns. M: molecular mass protein marker.

The amplified coding sequences, excluding the signal peptide tags, were cloned and expressed as full-length proteins in *E. coli*. Gene locus, protein reference number, given name, sequences of primers used for PCR amplifications and molecular mass of recombinants are depicted in Table 1. The recombinant proteins were purified by nickel affinity chromatography, and an aliquot of each protein was analyzed by SDS-PAGE (Fig. 1). All purified proteins were represented by major bands, although in some cases such as Lsa27 and MPL21, other less intense protein bands were also observed. Structural integrity of the purified proteins not previously published, LipL40, MPL36, rLIC12922 and rLIC12238, was assessed by CD spectroscopy. The minima at 208 and 222 nm, and the maximum at 192 nm in the CD spectrum showed the high α-helical secondary structure content of the recombinant proteins LipL40, MPL36 and rLIC12922, while rLIC12238 showed a predominant signal of β-strands, with minimum ellipticity around 215 nm (data not shown).

### Cellular localization of the recombinant proteins

**Figure 2:**
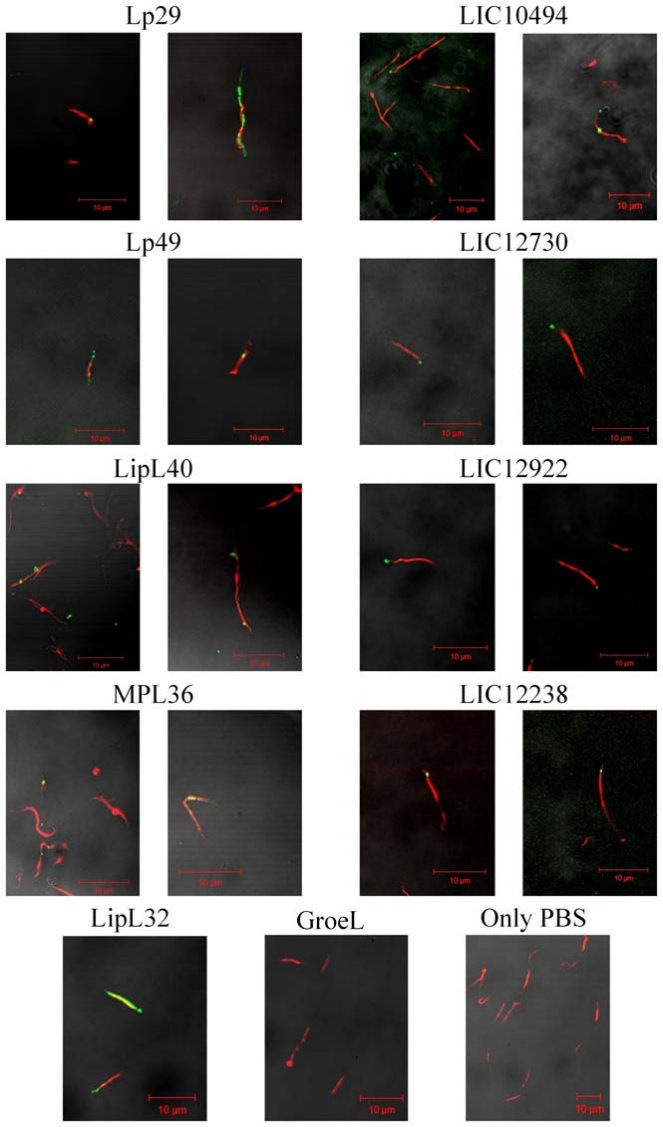
Cellular localization of proteins in *L. interrogans* by L-IFA. Protein recognition by L-IFA. Proteins in live *L. interrogans* serovar Copenhageni isolates were recognized by FITC-conjugated antibodies against Lp29, Lp49, LipL40, MPL36, LIC10494, LIC12730, LIC12922, LIC12238 and LipL32 (surface-exposed lipoprotein, positive control), but not against GroEL (a protoplasmic cylinder marker) and PBS (negative control) under confocal microscopy. The leptospires were identified using the DNA counterstain propidium iodide. Panels are composite images of propidium iodide- and FITC-stained slides. Magnification, 600×.

To determine whether the proteins not previously characterized, namely Lp29, Lp49, LipL40, MPL36, rLIC10494, rLIC12730, rLIC12922 and rLIC12238, are surface-exposed proteins, we set out to analyze the corresponding protein location on the bacteria using living *L. interrogans* serovar Copenhageni cells and the liquid-phase immunofluorescence method. Leptospires were visualized by propidium iodide staining followed by protein detection with polyclonal mouse antiserum raised against the protein in the presence of anti-mouse IgG antibodies conjugated to FITC. The localization of the protein-green light lying on the leptospires was achieved by superimposing the two fields, and the results obtained are shown in Fig. 2. For each protein, two fields are depicted where we can see, in general, green fluorescence spots along the bacteria. An intense green fluorescence could be observed for LipL32, a well-known major outer membrane protein of *Leptospira*, used as a positive control [38], but not with GroEL, a protoplasmic-cylinder marker used as a negative control [39]. The PBS control showed only red fluorescence of leptospires stained with propidium iodide.

### Recombinant leptospiral proteins bind human plasminogen

**Figure 3:**
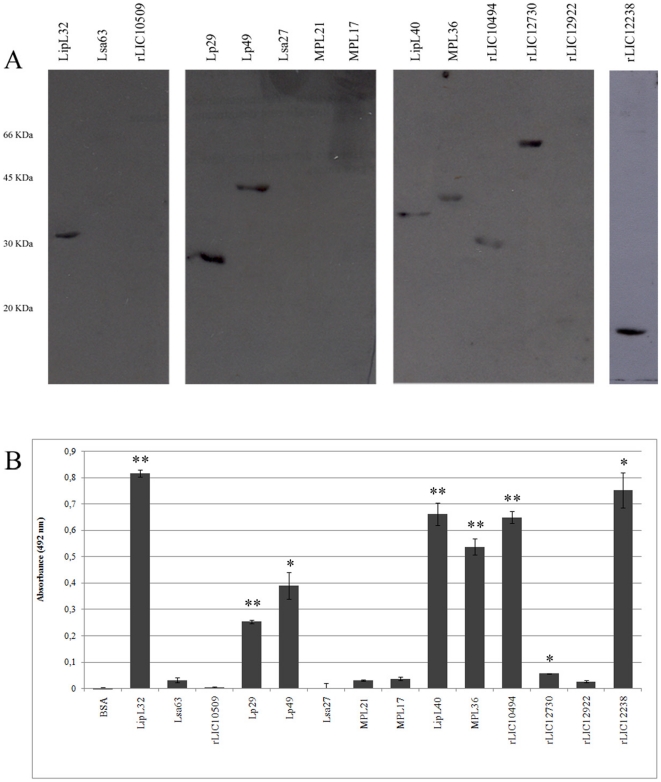
Binding of leptospiral recombinant proteins to human plasminogen. **A.** The proteins were electrophoresed in 12% SDS-PAGE gels and transferred to nitrocellulose membranes, followed by overnight incubation with 3 µg/mL PLG; after washing, the membrane was incubated with mouse anti-human PLG (1∶750), followed by incubation with anti-mouse IgG (1∶5,000). Protein reactivity was revealed by ECL reagent/exposure to X-ray films. **B.** The recombinant proteins LipL32, Lsa63, rLIC10509, Lp29, Lp49, Lsa27, MPL21, MPL17, LipL40, MPL36, rLIC10494, rLIC12730, rLIC12922 and rLIC12238 were coated onto 96-well ELISA plates (10 µg/ml) and allowed to interact with purified human PLG (10 µg/ml). BSA was used as a negative control for nonspecific binding. Binding was detected and quantified by specific antibodies. Bars represent the mean absorbance values at 492 nm ± the standard deviation of four replicates for each protein and are representative of three independent experiments. * *P*<0.001; ** *P*<0.0001.

We have reported that *L. interrogans* binds PLG and that several proteins could act as receptors [21]. These data prompted us to investigate whether the selected surface-exposed proteins are capable of binding human PLG *in vitro*. We have employed Western blotting assay as a first screening to identify PLG-binding proteins. The results show that the proteins LipL32, Lp29, Lp49, LipL40, MLP36, rLIC10494, rLIC12730 and rLIC12238 were interacting with PLG, while no binding was seen with Lsa63, Lsa27, rLIC10509, MPL21, MPL17, rLIC12730 and rLIC12922 (Fig. 3A). In addition, the data clearly indicate that binding only occur with the expected molecular mass corresponding to the recombinant proteins (Fig. 3A). The binding of the proteins to PLG was also evaluated by ELISA. The fourteen recombinant leptospiral proteins, and BSA, used as negative control, were individually immobilized onto 96-wells plates, incubated with human PLG and the results obtained from three independent experiments are shown in Fig. 3B. Proteins LipL32, Lp29, Lp49, LipL40, MLP36, rLIC10494 and rLIC12238 presented statistically significant binding to PLG (*P<0.001 and **P<0.0001), while proteins, Lsa63, Lsa27, rLIC10509, MPL21, MPL17, rLIC12730, rLIC12922 and BSA did not show significant binding activity (Fig. 3B). The ELISA data are in agreement with the one obtained with Western blotting except for rLIC12730 and rLIC12238 that show discrepancy in binding with the two methodologies. The rLIC12730 depicted a stronger binding by Western blotting when compared to ELISA data, while rLIC12238 showed an opposite behavior (Fig. 3A, 3B).

### Comparison of encoding sequences of the detected leptospiral plasminogen-binding proteins and those of published plasminogen-binding proteins

To investigate whether the identified PLG-binding proteins showed sequence similarity with other published spirochetal PLG-binding proteins [40], [41], [42], [43], [44], we carried out sequence alignment analysis using the Clustal X program and a tree-display NJ plot [45], [46]. The constructed dendrogram showed that leptospiral PLG-binding proteins do not display sequence similarity or share conserved domains with other reported spirochetal PLG-binding proteins (data not shown).

### Role of lysine residues in the plasminogen binding activity

**Figure 4:**
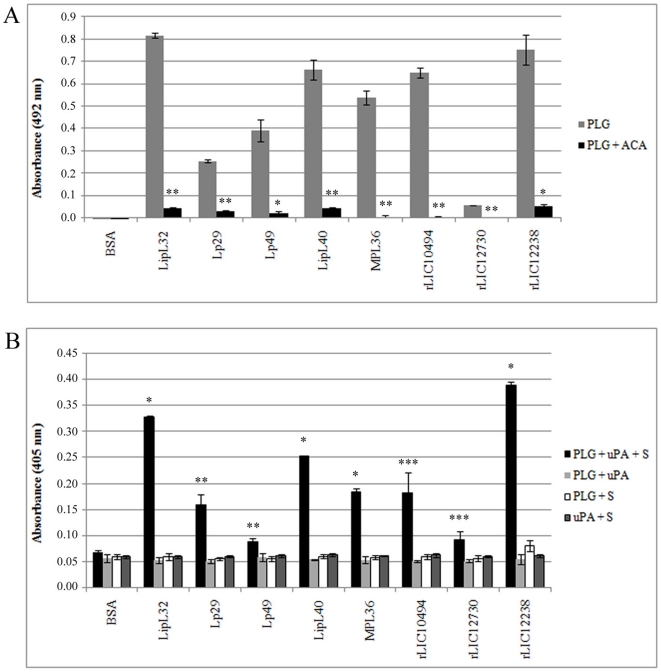
Role of lysine residues in recombinant protein-PLG interaction and activation to enzymatically active plasmin. **A:** Binding of the immobilized proteins LipL32, Lp29, Lp49, LipL40, MPL36, rLIC10494, rLIC12730 and rLIC12238 (10 µg/ml) to PLG (10 µg/ml) was carried out in the presence (PLG+ACA) or absence (PLG) of the lysine analogue 6-aminocaproic acid. BSA was used as a negative control for nonspecific binding. The bound PLG was detected and quantified by specific antibodies. Bars represent the mean absorbance at 492 nm ± the standard deviation of four replicates for each sample and are representative of two independent experiments. Statistically significant differences are shown by * (*P*<0.001) and ** (*P*<0.0001). **B:** Cleavage of specific plasmin substrate by PLG bound to recombinant proteins was assayed by modified ELISA as immobilized proteins received the following treatment: PLG+uPA+specific plasmin substrate (PLG+uPA+S), or controls lacking one of the three components (PLG+uPA; PLG+S; uPA+S). BSA was employed as a negative control. Bars represent the mean absorbance values at 405 nm, as a measure of relative substrate cleavage, ± the standard deviation of four replicates for each experimental group and are representative of two independent experiments. Statistically significant differences are shown by * (*P*<0.00001), ** (*P*<0.005) and *** (*P*<0.05).

It is known that PLG kringle domains frequently mediate interactions with lysine residues of the bacterial receptors [20]. These domains participate in the binding of PLG with intact live *L. interrogans* serovar Copenhageni strain L1–130, since the derivative and analogue of lysine, ACA, almost totally inhibited binding [21]. Sequence analysis showed that the native PLG binding proteins, LipL32, Lp29, Lp49, LipL40, MPL36, LIC10494, LIC12238 and LIC12730, contain 9.6, 9.3, 10.7, 7.8, 10.6, 9.1, 8.8 and 5.5% lysine residues, respectively. Based on these findings, the participation of lysine residues in the binding of the recombinant proteins was evaluated by the addition of ACA to the assay. As depicted in Fig. 4A, when 2 mM ACA was added to the reaction, the binding of the proteins to PLG was almost completely abolished.

### Activation of plasminogen-bound proteins

PLG bound to the surface of *L. interrogans* is converted to enzymatically active plasmin by the addition of activator [21]. To determine if PLG bound to recombinant proteins can acquire proteolytic activity, 96-well plates were coated with the test proteins, blocked, and then incubated with PLG. Unbound PLG was washed away and the uPA-type PLG activator was added together with a plasmin-specific chromogenic substrate. The reaction was carried out overnight and the plasmin activity was evaluated by measuring the cleavage of the plasmin-specific chromogenic substrate (absorbance at 405 nm). As shown in Fig. 4B, the PLG captured by the proteins could be converted into plasmin, as demonstrated indirectly by specific proteolytic activity. The negative control BSA did not bind PLG (see Fig. 4) and did not show any proteolytic activity, as with the controls lacking PLG, uPA or the chromogenic substrate.

### Characterization of the binding of recombinant proteins to PLG

**Figure 5:**
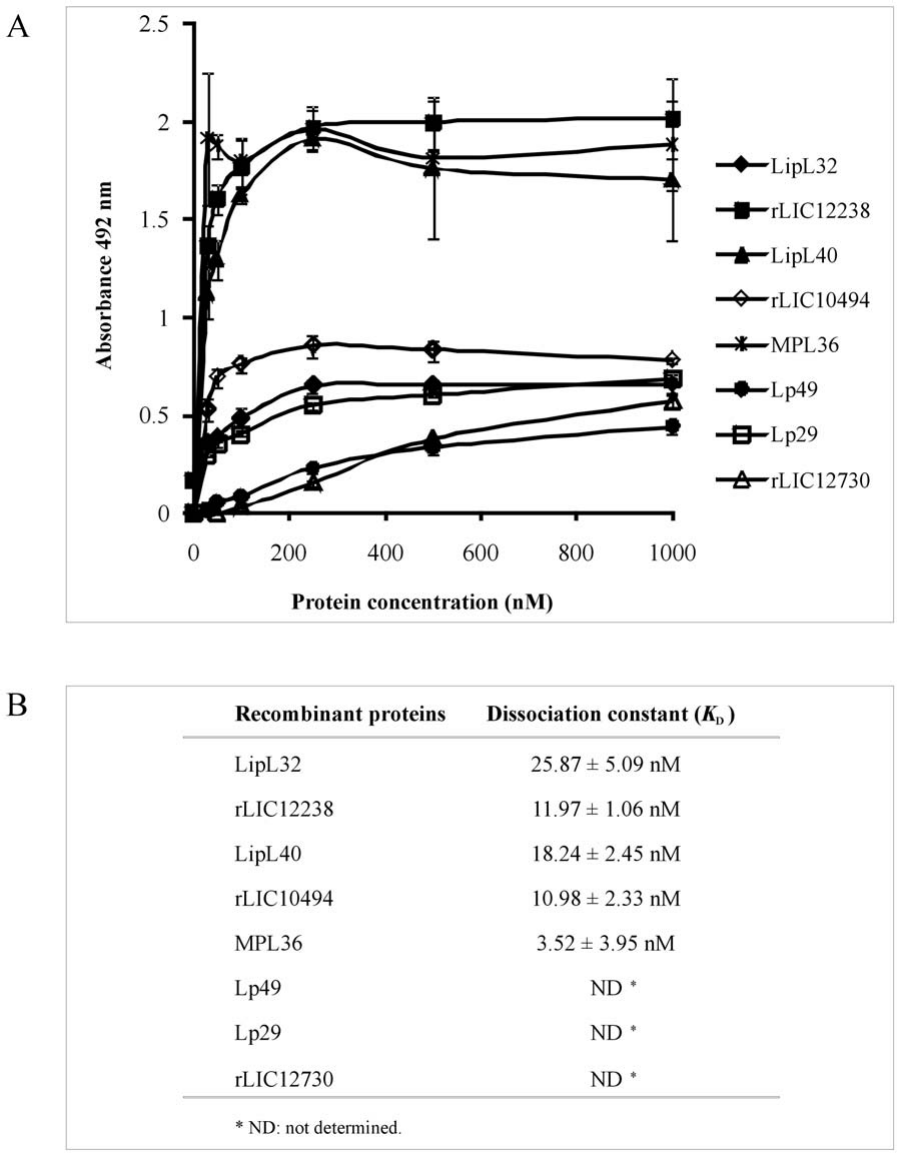
Characterization of recombinant proteins binding to PLG. A. PLG (10 µg/ml) was immobilized in 96-well ELISA plates, and each recombinant protein at 0 to 1,000 nM was added for interaction. The binding was detected using antiserum raised in mice against each protein at appropriate dilutions (1∶4,000 for LipL32; 1∶5,000 for rLIC12238, LipL40 and MPL36; 1∶1,000 for Lp29 and Lp49; 1∶500 for rLIC12730), followed by horseradish peroxidase-conjugated anti-mouse IgG. Data represent the mean absorbance values ± the standard deviation of six replicates for each experimental group. The results are representative of two independent experiments. B. The dissociation constant (*K*_D_) was calculated based on ELISA data for the recombinant proteins that reached equilibrium up to a concentration of 1,000 nM.

The interactions between the recombinant proteins and PLG were assessed on a quantitative basis as illustrated in Fig. 5A. Dose-dependent and saturable binding was observed when increasing concentrations (0 to 1,000 nM) of the recombinant proteins MPL36, LipL40, LipL32, rLIC10494 and rLIC12238 were allowed to individually adhere to a fixed PLG amount (1 µg). For the proteins Lp29, Lp49 and rLIC12730, the saturation level was not reached, even at the highest concentration tested (1,000 nM). Based on the ELISA data, the calculated dissociation equilibrium constants (*K*_D_) for the recombinant proteins with PLG are depicted in Fig. 5B; for the ones that reached equilibrium, the highest and the lowest *K*_D_ values were for LipL32 (25.87±5.09 nM) and MPL36 (3.52±3.95 nM), respectively.

### Effects of salt and heparin on LipL32- and rLIC12238 -plasminogen interactions

**Figure 6:**
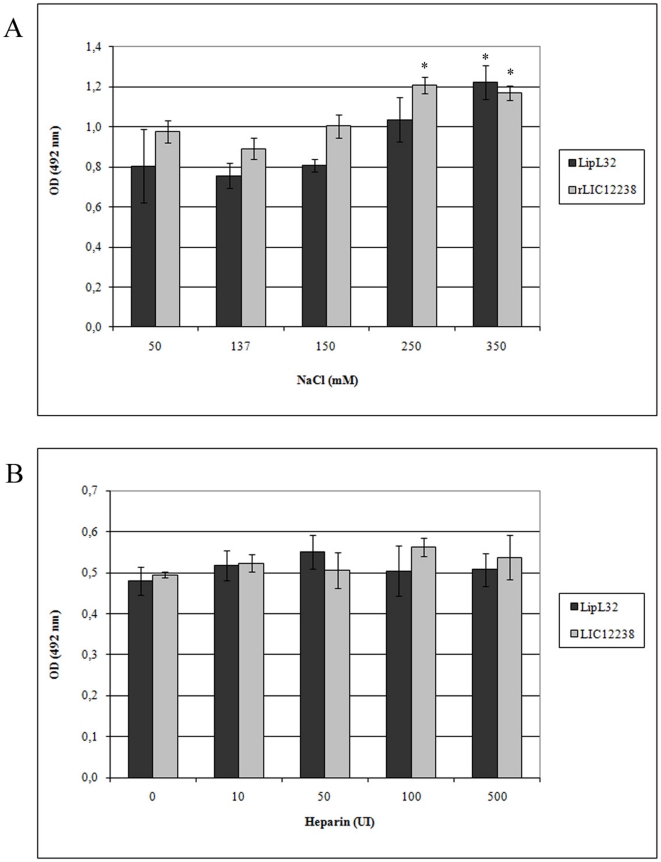
Effects of ionic strength and heparin in PLG binding by LipL32 and rLIC12238. Binding of LipL32 and rLIC12238 (10 µg/ml) to immobilized PLG (10 µg/ml) was performed with the addition of increasing concentrations of NaCl (0 to 350 mM) (A) or heparin (0 to 500 IU) (B). The detection of bound-PLG was assayed by specific antibodies. Bars represent the mean absorbance values ± the standard deviation of four replicates for each experimental group and are representative of two independent experiments. The data show statistically significant differences in **A**, shown by * (*P*<0.05), but not in **B**.

As the proteins LipL32 and rLIC12238 showed the highest PLG binding and plasmin activity (Fig. 3B and Fig. 4B), they were chosen for further studies. To verify whether binding of these recombinant proteins to PLG is affected by added Cl^−^ [47] or negatively charged heparin [48], binding assays were performed in the presence of different concentrations of NaCl or heparin. As shown in Fig. 6A, the increase in Cl anions to physiological conditions did not affect binding, and interference was only detected at a higher salt concentration, 350 mM, for both proteins. Similarly, the addition of heparin (up to 500 IU) did not have any significant effect on the interaction of the proteins with PLG (Fig. 6B).

### Collagen type IV, but not plasma fibronectin, competes with LipL32 for binding to PLG

**Figure 7:**
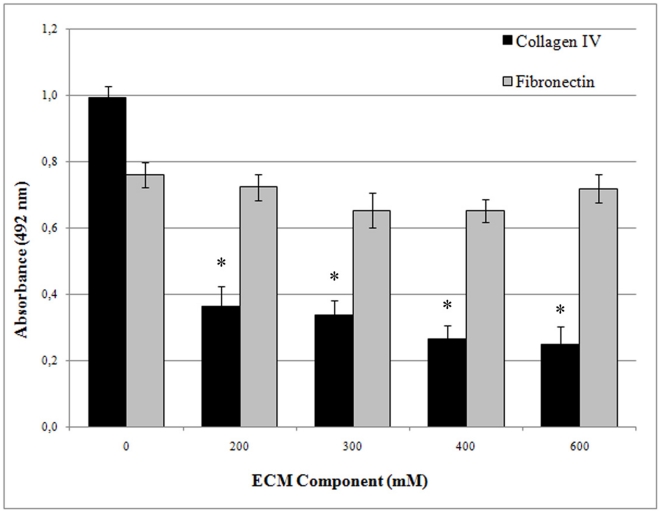
Effect of extracellular matrix macromolecules on PLG binding by LipL32. Binding of LipL32 (10 µg/ml) to immobilized PLG (10 µg/ml) was performed with the addition of increasing concentrations of plasma fibronectin or collagen type IV (0 to 600 mM). The detection of bound-PLG was assayed by specific antibodies. Bars represent the mean absorbance values ± the standard deviation of four replicates for each condition and are representative of two independent experiments. Results of statistically significant interference with binding in comparison with the positive control (no addition of purified ECM macromolecules) are shown: *P*<0.0003 (*).

LipL32 has been previously demonstrated to be a leptospiral adhesin capable of binding collagen type IV and plasma fibronectin [15], [16]. To see if the extracellular matrix components compete with LipL32 for the binding site of PLG or interfere somehow in the interaction with PLG, the binding assay was performed in the presence of increasing concentrations of collagen type IV or plasma fibronectin. The results show that plasma fibronectin had no effect on the binding of LipL32 to PLG (Fig. 7). On the other hand, the addition of collagen type IV decreased LipL32 binding to PLG in a dose-dependent fashion (Fig. 7), suggesting that both molecules compete for the same binding site.

## Discussion

The interaction of the human plasminogen system has been suggested to be a feature that significantly contributes to the virulence of many bacterial pathogens by facilitating the initial anchoring to the endothelium [20]. Through capturing the host PLG on its surface, followed by activation to enzymatically active plasmin, the bacteria are turned into proteolytic organisms, a characteristic that has been shown to contribute to the degradation of ECM components, tissue penetration and invasion. In our previous work, we showed that leptospires are capable of capturing PLG on its outer surface and that the addition of exogenous activator converted it into enzymatically active plasmin capable of degrading ECM fibronectin [21]. In addition, multiple PLG-binding proteins present in the *L. interrogans* serovar Copenhageni whole cell lysates were detected, but remained unidentified [21]. Moreover, as expected, our data suggest the involvement of outer membrane proteins in the binding. Indeed, Verma and colleagues [27] have recently demonstrated that LenA, a surface leptospiral protein [14] is a bacterial receptor for human plasminogen. The availability of several putative surface-exposed recombinant proteins of *L. interrogans* in our laboratory prompted us to investigate the ability of these proteins to interact with PLG *in vitro*, in order to reveal and extend the array of plasminogen receptors in *Leptospira*.

The fourteen proteins selected for the screening of PLG binding were cloned and expressed in *E. coli* as full-length recombinant proteins. The purified proteins exhibited major bands in SDS-PAGE making them suitable for PLG binding assays. The structural integrity of the unpublished purified proteins was attested by CD spectroscopy revealing a mixture of α-helix and β–strand structures. The other recombinant proteins had their CD spectra confirmed and were similar to the ones previously reported [11], [12], [19], [30], [31], [33]. The cellular localization of the proteins Lp29, Lp49, LipL40, MPL36, rLIC10494, rLIC12730, rLIC12922 and rLIC12238, was shown to be the cell surface, similar to findings for the previously published LipL32 [28], Lsa63 [19], rLIC10509 [33], Lsa27 [11], MPL21 and MPL17 [31].

Several invasive gram-positive and gram-negative bacteria have been shown to interact with the host PLG system [20], [49], [50], [51], [52], a phenomenon that has also been observed with viruses [53], [54], [55] and parasites [56], either by expression of PLG receptors or stimulation of host activators. In the case of spirochetes, the plasminogen activation system was studied with several species of *Borrelia* with *Treponema denticola* and suggested to have an important role during infection [22], [23], [24], [25], [26].

The human plasminogen heavy chain contains five triple-disulfide-linked peptide regions of ∼80 amino acids, called kringles [57]. The process of binding to bacterial receptors on the cell surface has been shown for several pathogens to be mediated by the PLG kringle domains through the lysine-binding sites present [20]. Similarly, *Borrelia* [24] and *Leptospira* [21] also bind PLG via kringle domains. We have identified eight leptospiral plasminogen-binding proteins: LipL32 [28], [32], rLIC10494, rLIC12730 [12], Lp29, Lp49 [30] and LipL40 and MPL36 [29] and one novel protein rLIC12238. The specificity of the binding of these recombinant proteins was attested by Western blotting. Interestingly, two proteins showed disparate binding intensity by Western blotting and ELISA. This could be explained by the fact that ELISA is a quantitative method but Western blotting is not. In addition, due to the denaturing condition of this latter method, it is possible that the exposure of some epitopes led to better binding for rLIC12730, while the inverse situation was seen with rLIC12238 which apparently needs a more native condition for PLG binding.

Plasminogen-binding proteins of *B. burgdorferi*, including the outer-surface lipoprotein OspA, have been identified [41]. Recently, Brissette and coworkers [44] identified three proteins of *B. burgdorferi*, ErpP, ErpA and ErpC, as being PLG-binding proteins. Interestingly, an evaluation of the primary sequence of spirochete PLG-binding proteins show that the proteins do not share similarity, with the exception of the Erp family protein of *B. burgdorferi* [44], BbCRASP-1 of *B. hermsii* [43] and HcpA of *B. recurrentis* [40].

Our data with the PLG-binding proteins point out to a primary role for lysine residues in plasminogen binding, as the lysine analog 6-aminocaproic acid (ACA) significantly inhibited binding [21], [27], [58]. However, no correlation between the number of lysine residues and PLG binding was detected. Indeed, the highest percentage of lysine residues was identified in Lsa63 [19], a protein that did not bind PLG. Moreover, lysine residue position does not seem to correlate with binding because they were distributed among all the protein sequences studied. This suggests that protein conformation and steric hindrance should be important factors in the binding of the protein/receptor to PLG. Plasminogen-binding proteins were able to generate active plasmin in the presence of urokinase-type PLG activator, as measured by the cleavage of plasmin-specific chromogenic substrate.

The recombinant proteins bind to PLG in a concentration-dependent and saturable fashion, indicating the specificity of the binding, for the proteins LipL32, LipL40, MPL36, rLIC12238 and rLIC10494. The calculated dissociation equilibrium constants (*K*_D_) for the recombinant proteins with PLG varied from 25.87±5.09 nM for LipL32 to 3.52±3.95 nM for MPL36. The plasminogen interaction/activation has been shown to be suppressed by the presence of Cl anions [47], while there is evidence that heparin dextran derivatives could contribute to the regulation of plasmin activity, not only by impeding plasmin generation, as a result of their binding to plasminogen but also by directly affecting the catalytic activity of the enzyme [48]. The addition of Cl^−^ did not inhibit LipL32 or rLIC12238-PLG interactions, showing in fact, a positive effect with increasing sodium chloride concentration. The presence of heparin had no influence on the binding of either protein to PLG. Similar results were obtained by Brissette and coworkers [44] studying the effect of both compounds on the interactions of ErpP recombinant protein of *B. burgdorferi* and plasminogen. Furthermore, Verma et al. [27] showed that heparin had no effect on LenA-PLG interactions but that the addition of salt led to an increase in binding.

The identified PLG-binding proteins Lp29, Lp49, LipL40 and MPL36 have been previously reported to be reactive with sera from leptospirosis patients [29], [30] while rLIC12238 and rLIC10494 react with sera from hamsters infected with *Leptospira* (Nascimento, AL et al., unpublished results). These data suggest that the proteins are expressed and exposed during the course of infection. We confirmed here the localization of these four proteins and presented their PLG-binding properties. Taken together, our results implicate that these proteins play a possible role in virulence.

The protein LipL32 is a major outer membrane antigen expressed during mammalian infection [16], [28]. It has been shown that LipL32 is recognized by patients' sera [15], [59] and has the ability to bind to ECM macromolecules [15], activities that suggest a role in the pathogenesis of leptospires. Intriguingly, it has also been demonstrated that LipL32 is not essential for either acute or chronic infection with *L. interrogans* [60]. Here, we provide another activity for LipL32, as a PLG-binding protein, giving further support to its importance during infection. Additionally, our results suggest that collagen type IV and PLG interact with the same site of LipL32, a situation that does not occur with plasma fibronectin. The dual activity of LipL32 in binding PLG and ECM macromolecules together with our previous findings of fibronectin degradation by plasmin-coated leptospires [21], may have implications on the mechanisms of the invasion process.

In conclusion, we have identified and characterized eight proteins as being PLG-binding proteins of *Leptospira*. There is evidence that at least five of them (LipL32, Lp29, Lp49, LipL40 and MPL36) are expressed during natural infection [28], [29], [30]. PLG-binding/activation through the proteins/receptors on the surface of *Leptospira* could help the bacteria to specifically overcome tissue barriers, facilitate invasion and colonize mammalian tissues. We believe that these studies provide new insights into leptospiral pathogenesis and infection.

## Materials and Methods

### Bioinformatics characterization of the proteins

Predicted coding sequences (CDSs ) were analyzed as their cellular localization predictions by PSORT program, http://psort.nibb.ac.jp [61], [62]. The web servers SMART, http://smart.embl-heidelberg.de/ [63], [64], PFAM, http://www.sanger.ac.uk/Software/Pfam/ [65], and LipoP, http://www.cbs.dtu.dk/services/LipoP/ [66] were used to search for predicted functional and structural domains within the amino acid sequences of the CDSs.

### Cloning, expression and purification of recombinant proteins

Amplification of the CDSs was performed by PCR from *L. interrogans* serovar Copenhageni genomic DNA using complementary primer pairs. The gene sequences were amplified without the signal peptide tag, predicted by SignalP (http://www.cbs.dtu.dk/services/SignalP/ ). The final constructs were verified by DNA sequencing on an ABI Prism 3730_L sequencer (Seq- Wright, Houston, TX) with appropriate vector-specific T7 (F: TAATACGACTCACTATAGGG) and pAE (R:CAGCAGCCAACTCAGTTCCT) primers. Cloning, expression and purification of the recombinant proteins Lsa63, LIC10509, Lp29, Lp49 and Lsa27 have been previously described [11], [19], [30], [33]. The sequences coding for MPL21, MPL17, MPL36, LipL40, LipL32, LIC10494, LIC12730 and LIC12922 were cloned into the *E. coli* Gateway cloning (pENTR) and expression (pDEST17) system (Invitrogen), according to the protocol described elsewhere [29]. The expression and purification of MPL21 and MPL17 were performed as reported elsewhere [31], and rLIC10494 and rLIC12730, as previously described [12]. The recombinant proteins MPL36 and LipL40 were purified from *E. coli* BL21 (DE3) and *E. coli* BL21-SI, respectively. Bacteria were grown in Luria-Bertani (LB) broth with or without NaCl, at 37° or 30°C, and at log-phase they were induced by 1 mM isopropyl-β-D-thiogalactopyranoside (IPTG) or 3 mM NaCl for 3 h at 37°C or 30°C, respectively. The bacteria cell lysates were cleared by centrifugation at 3,000×g for 15 min, and the supernatants were loaded onto Ni^2+-^charged Sepharose beads. The beads were washed with increasing concentrations of imidazole (20 to 60 mM) and the proteins eluted with 0.5 M imidazole. The proteins LipL32 and rLIC12922 were expressed in *E. coli* BL21-SI for 3 h at 30°C with the addition of 150 mM NaCl to the log-phase culture grown in LB broth without NaCl. The proteins were expressed in the soluble form, as the above protocol described for the proteins MPL36 and LipL40. The LIC12238 CDS was cloned into the pGEM-T-Easy vector (Promega), and transferred into the pAE expression vector [67]. The construct was transformed into *E. coli* BL21-SI, and the log-phase culture, grown at 30°C, was induced by incubation in the presence of 300 mM NaCl for 3 h. The protein rLIC12238 was expressed in the soluble form and purified as described above.

### Circular dichroism spectroscopy

Purified recombinant proteins were dialyzed against sodium phosphate buffer (pH 7.4). Circular dichroism (CD) spectroscopy measurements were performed at 20°C using a Jasco J-810 spectropolarimeter (Japan Spectroscopic, Tokyo) equipped with a Peltier unit for temperature control. Far-UV CD spectra were measured using a 1 mm-path length cell at 0.5 nm intervals. The spectra were presented as an average of five scans recorded from 180 to 260 nm. The molar ellipticity (Φ) is expressed in deg.cm^2^.dmol^−1^.

### Antisera production against recombinant proteins

The recombinant proteins were adsorbed onto 10% (vol/vol) of Alhydrogel (2% Al (OH)_3_, Brenntag Biosector, Denmark), used as adjuvant. Each recombinant protein (10 µg) was subcutaneously injected in female BALB/c mice (4–6 weeks old). Two subsequent booster injections were given at two-week intervals with the same protein preparations. Negative-control mice were injected with PBS/Alhydrogel. Two weeks after each immunization, the mice were bled from the retro-orbital plexus, and the pooled sera were analyzed by enzyme-linked immunosorbent assay for the determination of antibody titers. All animal studies were approved by the Ethics Committee of Instituto Butantan, under protocol 506/08, São Paulo, SP, Brazil.

### Liquid-phase immunofluorescence assay (L-IFA)

The localization of the recombinant proteins by L-IFA was performed as previously described [31]. In brief, 2.5-ml suspensions of live leptospires (*L. interrogans* serovar Copenhageni strain M 20, routinely cultured as described elsewhere [21] were harvested at 10,000 rpm for 15 min, washed twice with PBS (with 50 mM NaCl), resuspended in 200 µl of PBS with 6 µg/ml propidium iodide to stain the nuclei, and incubated for 45 min at 37°C. After incubation, the leptospires were gently washed with PBS and incubated for 30 min at 4°C with polyclonal mouse antiserum against each recombinant protein or GroEL at a 1∶50 dilution. The leptospires were washed and incubated with goat anti-mouse IgG antibodies conjugated to fluorescein isothiocyanate (FITC, Sigma) at a dilution 1∶50 for 30 min at 4°C. After incubation with secondary antibody, the leptospires were washed and resuspended in PBS-antifading solution (ProLong Gold, Molecular Probes). The immunofluorescently labeled leptospires were examined with the use of a confocal LSM 510 META immunofluorescence microscope (Zeiss, Germany).

### Plasminogen binding

The binding of the recombinant proteins to PLG was evaluated by a modified ELISA as follows: 96-well plates (Costar High Binding, Corning) were coated overnight in PBS at 4°C with 100 µl of 10 µg/ml of the recombinant proteins or bovine serum albumin (BSA) as negative control. Plates were washed once with PBS supplemented with 0.05% (vol/vol) Tween 20 (PBS-T) and blocked for 2 h at 37°C with PBS with 10% (wt/vol) non-fat dry milk. The blocking solution was discarded and 100 µl of 10 µg/ml human plasminogen in PBS was incubated for 2 h at 37°C. Wells were washed four times with PBS-T and incubated for 1 h at 37°C with mouse anti-human plasminogen (Sigma-Aldrich) (1∶4,000 in PBS). Plates were washed again and incubated with horseradish peroxidase-conjugated anti-mouse immunoglobulin G (IgG), diluted 1∶5,000 in PBS. After three washings, 100 µl/well of 1 mg/ml *o*-phenylenediamine (OPD) plus 1 µl/ml H_2_O_2_ in citrate phosphate buffer (pH 5.0) were added. The reactions were carried out for 5 min and stopped by the addition of 50 µl/well of H_2_SO_4_ (2 N). Readings were taken at 492 nm.

### Effects of salt and heparin on recombinant proteins-plasminogen interactions

To assess the role of ionic interactions or of heparin-binding domains in LipL32 or rLIC12238 binding to plasminogen, increasing concentrations of NaCl (0–350 mM) or heparin (0–500 IU) (Roche) were added to the PBS-based buffer with plasminogen to the recombinant protein-coated wells. In both experiments, the detection of bound-plasminogen was performed as described above.

### SDS-PAGE and affinity blotting

The purified recombinant proteins were electrophoresed in 12% SDS-PAGE gels and transferred to nitrocellulose membranes (Hybond-ECL, GE Healthcare) in semi-dry equipment. The membranes were blocked with 5% BSA for 2 h at 37°C, washed three times (10 min for each wash) with PBS-0.5% Tween-20 solution and incubated overnight with 3 µg/mL PLG 4°C, followed by 2h incubation at room temperature. The membranes were then washed three times and incubated with mouse anti-human PLG (1∶750) for 3 h at room temperature, followed by three more washings and 1 h incubation at room temperature with anti-mouse IgG (1∶5,000). The membranes were washed and the protein's reactivity was revealed using the ECL reagent (GE Healthcare) with subsequent exposure to X-ray films.

### Characterization of the binding

To determine the role of lysines in plasminogen-recombinant protein interactions, the lysine analog 6-aminocaproic acid (ACA) (Sigma-Aldrich) was added together with plasminogen at a final concentration of 2 mM to the recombinant protein-coated wells. To assess the role of ionic interactions or of heparin-binding domains in LipL32 or rLIC12238 binding to plasminogen, increasing concentrations of NaCl (0–350 mM) or heparin (0–500 IU) (Roche) were added to the PBS-based buffer with plasminogen which was then added to the recombinant protein-coated wells. In both experiments, the detection of bound-plasminogen was performed as described above.

### Plasmin enzymatic activity assay

First, 96-well ELISA plates were coated overnight with 10 µg/ml recombinant proteins or BSA in PBS at 4°C. Plates were then washed once with PBS-T and blocked with PBS with 10% (wt/vol) non-fat dry milk for 2 h at 37°C. The blocking solution was discarded and 100 µl/well of 10 µg/ml human plasminogen was added, followed by incubation for 2 h at 37°C. Wells were washed three times with PBS-T, and then 4 ng/well of human uPA (Sigma-Aldrich) were added. Subsequently, 100 µl/well of the plasmin-specific substrate D-valyl-leucyl-lysine-*p*-nitroanilide dihydrochloride (Sigma-Aldrich) was added at a final concentration of 0.4 mM in PBS. Plates were incubated overnight at 37°C and substrate was measured by taking readings at 405 nm.

### Dose-response curves

First, 96-well ELISA plates were coated overnight in PBS at 4°C with 100 µl of 10 µg/ml plasminogen. Plates were then blocked and increasing concentrations of the purified recombinant proteins (0–1 µM) were added (100 µl/well in PBS). The assessment of bound proteins was performed by incubation for 1 h at 37°C with the antiserum raised against each protein at appropriate dilutions (1∶4,000 for LipL32; 1∶5,000 for rLIC12238, LipL40 and MPL36; 1∶1,000 for Lp29 and Lp49; 1∶500 for rLIC12730), followed by horseradish peroxidase-conjugated anti-mouse IgG (Sigma) (1∶10,000 in PBS). The binding was evaluated by the peroxidase substrate OPD and readings were taken at 492 nm. The ELISA data were used to calculate the dissociation constant (*K*_D_) according to the method described by Pathirana et al. [68] and Lin et al. [69], based on the equation: A = Amax [protein]/(*K*_D_+[protein]), where A is the absorbance at a given protein concentration, Amax is the maximum absorbance for the ELISA plate reader (equilibrium), [protein] is the protein concentration and *K*_D_ is the dissociation equilibrium constant for a given absorbance at a given protein concentration (ELISA data point).

### Interference of collagen type IV and plasma fibronectin with PLG binding by LipL32

First, 96-well plates were coated overnight at 4°C with 100 µl of 10 µg/ml recombinant LipL32 in PBS, washed once and blocked with 10% (w/v) non-fat dry milk for 2 h at 37°C. The plates were then incubated for 2 h at 37°C with 200 nM PLG together with increasing concentrations of collagen type IV (Sigma-Aldrich) or plasma fibronectin (Sigma-Aldrich) (both from 0 to 600 nM). After four washings, PLG binding was quantified by specific antibodies as described above.

## References

[1] Haake DA, Dundoo M, Cader R, Kubak BM, Hartskeerl RA (2002). Leptospirosis, water sports, and chemoprophylaxis.. Clin Infect Dis.

[2] Bharti AR, Nally JE, Ricaldi JN, Matthias MA, Diaz MM (2003). Leptospirosis: a zoonotic disease of global importance.. Lancet Infect Dis.

[3] Faine S, Adler B, Bolin C, Perolat P (1999). Leptospira and Leptospirosis. Second ed.

[4] Levett PN (2001). Leptospirosis.. Clin Microbiol Rev.

[5] Vinetz JM (2001). Leptospirosis.. Curr Opin Infect Dis.

[6] Plank R, Dean D (2000). Overview of the epidemiology, microbiology, and pathogenesis of Leptospira spp. in humans.. Microbes Infect.

[7] Matsunaga J, Barocchi MA, Croda J, Young TA, Sanchez Y (2003). Pathogenic Leptospira species express surface-exposed proteins belonging to the bacterial immunoglobulin superfamily.. Mol Microbiol.

[8] Ristow P, Bourhy P, da Cruz McBride FW, Figueira CP, Huerre M (2007). The OmpA-like protein Loa22 is essential for leptospiral virulence.. PLoS Pathog.

[9] Atzingen MV, Barbosa AS, De Brito T, Vasconcellos SA, de Morais ZM (2008). Lsa21, a novel leptospiral protein binding adhesive matrix molecules and present during human infection.. BMC Microbiol.

[10] Patti JM, Allen BL, McGavin MJ, Hook M (1994). MSCRAMM-mediated adherence of microorganisms to host tissues.. Annu Rev Microbiol.

[11] Longhi MT, Oliveira TR, Romero EC, Goncales AP, Morais ZM (2009). A novel identified protein of Leptospira interrogans mediates binding to laminin.. J Med Microbiol.

[12] Barbosa AS, Abreu PA, Neves FO, Atzingen MV, Watanabe MM (2006). A newly identified leptospiral adhesin mediates attachment to laminin.. Infect Immun.

[13] Choy HA, Kelley MM, Chen TL, Moller AK, Matsunaga J (2007). Physiological osmotic induction of Leptospira interrogans adhesion: LigA and LigB bind extracellular matrix proteins and fibrinogen.. Infect Immun.

[14] Stevenson B, Choy HA, Pinne M, Rotondi ML, Miller MC (2007). Leptospira interrogans endostatin-like outer membrane proteins bind host fibronectin, laminin and regulators of complement.. PLoS ONE.

[15] Hauk P, Macedo F, Romero EC, Vasconcellos SA, de Morais ZM (2008). In LipL32, the major leptospiral lipoprotein, the C terminus is the primary immunogenic domain and mediates interaction with collagen IV and plasma fibronectin.. Infect Immun.

[16] Hoke DE, Egan S, Cullen PA, Adler B (2008). LipL32 is an extracellular matrix-interacting protein of Leptospira spp. and Pseudoalteromonas tunicata.. Infect Immun.

[17] Atzingen MV, Gomez RM, Schattner M, Pretre G, Goncales AP (2009). Lp95, a novel leptospiral protein that binds extracellular matrix components and activates e-selectin on endothelial cells.. J Infect.

[18] Carvalho E, Barbosa AS, Gomez RM, Cianciarullo AM, Hauk P (2009). Leptospiral TlyC is an extracellular matrix-binding protein and does not present hemolysin activity.. FEBS Lett.

[19] Vieira ML, de Morais ZM, Goncales AP, Romero EC, Vasconcellos SA Lsa63, a newly identified surface protein of Leptospira interrogans binds laminin and collagen IV.. J Infect.

[20] Lahteenmaki K, Kuusela P, Korhonen TK (2001). Bacterial plasminogen activators and receptors.. FEMS Microbiol Rev.

[21] Vieira ML, Vasconcellos SA, Goncales AP, de Morais ZM, Nascimento AL (2009). Plasminogen acquisition and activation at the surface of leptospira species lead to fibronectin degradation.. Infect Immun.

[22] Nordstrand A, Shamaei-Tousi A, Ny A, Bergstrom S (2001). Delayed invasion of the kidney and brain by Borrelia crocidurae in plasminogen-deficient mice.. Infect Immun.

[23] Coleman JL, Gebbia JA, Piesman J, Degen JL, Bugge TH (1997). Plasminogen is required for efficient dissemination of B. burgdorferi in ticks and for enhancement of spirochetemia in mice.. Cell.

[24] Coleman JL, Sellati TJ, Testa JE, Kew RR, Furie MB (1995). Borrelia burgdorferi binds plasminogen, resulting in enhanced penetration of endothelial monolayers.. Infect Immun.

[25] Fenno JC, Tamura M, Hannam PM, Wong GW, Chan RA (2000). Identification of a Treponema denticola OppA homologue that binds host proteins present in the subgingival environment.. Infect Immun.

[26] Klempner MS, Noring R, Epstein MP, McCloud B, Rogers RA (1996). Binding of human urokinase type plasminogen activator and plasminogen to Borrelia species.. J Infect Dis.

[27] Verma A, Brissette CA, Bowman AA, Shah ST, Zipfel PF Leptospiral endostatin-like protein A (LenA) is a bacterial cell-surface receptor for human plasminogen.. Infect Immun.

[28] Haake DA, Chao G, Zuerner RL, Barnett JK, Barnett D (2000). The leptospiral major outer membrane protein LipL32 is a lipoprotein expressed during mammalian infection.. Infect Immun.

[29] Gamberini M, Gomez RM, Atzingen MV, Martins EA, Vasconcellos SA (2005). Whole-genome analysis of Leptospira interrogans to identify potential vaccine candidates against leptospirosis.. FEMS Microbiol Lett.

[30] Neves FO, Abreu PA, Vasconcellos SA, de Morais ZM, Romero EC (2007). Identification of a novel potential antigen for early-phase serodiagnosis of leptospirosis.. Arch Microbiol.

[31] Oliveira TR, Longhi MT, de Morais ZM, Romero EC, Blanco RM (2008). Evaluation of leptospiral recombinant antigens MPL17 and MPL21 for serological diagnosis of leptospirosis by enzyme-linked immunosorbent assays.. Clin Vaccine Immunol.

[32] Branger C, Sonrier C, Chatrenet B, Klonjkowski B, Ruvoen-Clouet N (2001). Identification of the hemolysis-associated protein 1 as a cross-protective immunogen of Leptospira interrogans by adenovirus-mediated vaccination.. Infect Immun.

[33] Gomez RM, Vieira ML, Schattner M, Malaver E, Watanabe MM (2008). Putative outer membrane proteins of Leptospira interrogans stimulate human umbilical vein endothelial cells (HUVECS) and express during infection.. Microb Pathog.

[34] Ren SX, Fu G, Jiang XG, Zeng R, Miao YG (2003). Unique physiological and pathogenic features of Leptospira interrogans revealed by whole-genome sequencing.. Nature.

[35] Nascimento AL, Verjovski-Almeida S, Van Sluys MA, Monteiro-Vitorello CB, Camargo LE (2004). Genome features of Leptospira interrogans serovar Copenhageni.. Braz J Med Biol Res.

[36] Bulach DM, Zuerner RL, Wilson P, Seemann T, McGrath A (2006). Genome reduction in Leptospira borgpetersenii reflects limited transmission potential.. Proc Natl Acad Sci U S A.

[37] Picardeau M, Bulach DM, Bouchier C, Zuerner RL, Zidane N (2008). Genome sequence of the saprophyte Leptospira biflexa provides insights into the evolution of Leptospira and the pathogenesis of leptospirosis.. PLoS ONE.

[38] Nally JE, Whitelegge JP, Bassilian S, Blanco DR, Lovett MA (2007). Characterization of the outer membrane proteome of Leptospira interrogans expressed during acute lethal infection.. Infect Immun.

[39] Haake DA, Matsunaga J (2002). Characterization of the leptospiral outer membrane and description of three novel leptospiral membrane proteins.. Infect Immun.

[40] Grosskinsky S, Schott M, Brenner C, Cutler SJ, Kraiczy P (2009). Borrelia recurrentis employs a novel multifunctional surface protein with anti-complement, anti-opsonic and invasive potential to escape innate immunity.. PLoS One.

[41] Fuchs H, Wallich R, Simon MM, Kramer MD (1994). The outer surface protein A of the spirochete Borrelia burgdorferi is a plasmin(ogen) receptor.. Proc Natl Acad Sci U S A.

[42] Lagal V, Portnoi D, Faure G, Postic D, Baranton G (2006). Borrelia burgdorferi sensu stricto invasiveness is correlated with OspC-plasminogen affinity.. Microbes Infect.

[43] Rossmann E, Kraiczy P, Herzberger P, Skerka C, Kirschfink M (2007). Dual binding specificity of a Borrelia hermsii-associated complement regulator-acquiring surface protein for factor H and plasminogen discloses a putative virulence factor of relapsing fever spirochetes.. J Immunol.

[44] Brissette CA, Haupt K, Barthel D, Cooley AE, Bowman A (2009). Borrelia burgdorferi infection-associated surface proteins ErpP, ErpA, and ErpC bind human plasminogen.. Infect Immun.

[45] Perriere G, Gouy M (1996). WWW-query: an on-line retrieval system for biological sequence banks.. Biochimie.

[46] Thompson JD, Gibson TJ, Plewniak F, Jeanmougin F, Higgins DG (1997). The CLUSTAL_X windows interface: flexible strategies for multiple sequence alignment aided by quality analysis tools.. Nucleic Acids Res.

[47] Urano T, Sator de Serrano V, Chibber BA, Castellino FJ (1987). The control of the urokinase-catalyzed activation of human glutamic acid 1-plasminogen by positive and negative effectors.. J Biol Chem.

[48] Ledoux D, Papy-Garcia D, Escartin Q, Sagot MA, Cao Y (2000). Human plasmin enzymatic activity is inhibited by chemically modified dextrans.. J Biol Chem.

[49] Degen JL, Bugge TH, Goguen JD (2007). Fibrin and fibrinolysis in infection and host defense.. J Thromb Haemost.

[50] Bergmann S, Hammerschmidt S (2007). Fibrinolysis and host response in bacterial infections.. Thromb Haemost.

[51] Coleman JL, Benach JL (1999). Use of the plasminogen activation system by microorganisms.. J Lab Clin Med.

[52] Sun H (2006). The interaction between pathogens and the host coagulation system.. Physiology (Bethesda).

[53] Goto H, Kawaoka Y (1998). A novel mechanism for the acquisition of virulence by a human influenza A virus.. Proc Natl Acad Sci U S A.

[54] Okumura Y, Yano M, Murakami M, Mori S, Towatari T (1999). The extracellular processing of HIV-1 envelope glycoprotein gp160 by human plasmin.. FEBS Lett.

[55] LeBouder F, Morello E, Rimmelzwaan GF, Bosse F, Pechoux C (2008). Annexin II incorporated into influenza virus particles supports virus replication by converting plasminogen into plasmin.. J Virol.

[56] Rojas M, Labrador I, Concepcion JL, Aldana E, Avilan L (2008). Characteristics of plasminogen binding to Trypanosoma cruzi epimastigotes.. Acta Trop.

[57] Castellino FJaP, A.V, Waisman D (2003). Plasminogen: structure, activation, and regulation.

[58] Crowe SD, Sievwright JK, Auld GC, Hoore NR, Gow NA (2003). *Candida albicans* binds human plasminogen identification of eight plasminogen-binding proteins.. Mol Microbiol.

[59] Flannery B, Costa D, Carvalho FP, Guerreiro H, Matsunaga J (2001). Evaluation of recombinant Leptospira antigen-based enzyme-linked immunosorbent assays for the serodiagnosis of leptospirosis.. J Clin Microbiol.

[60] Murray GL, Srikram A, Hoke DE, Wunder EA, Henry R (2009). Major surface protein LipL32 is not required for either acute or chronic infection with Leptospira interrogans.. Infect Immun.

[61] Nakai K, Horton P (1999). PSORT: a program for detecting sorting signals in proteins and predicting their subcellular localization.. Trends Biochem Sci.

[62] Nakai K, Kanehisa M (1991). Expert system for predicting protein localization sites in gram-negative bacteria.. Proteins.

[63] Letunic I, Copley RR, Pils B, Pinkert S, Schultz J (2006). SMART 5: domains in the context of genomes and networks.. Nucleic Acids Res.

[64] Schultz J, Milpetz F, Bork P, Ponting CP (1998). SMART, a simple modular architecture research tool: identification of signaling domains.. Proc Natl Acad Sci U S A.

[65] Finn RD, Mistry J, Schuster-Bockler B, Griffiths-Jones S, Hollich V (2006). Pfam: clans, web tools and services.. Nucleic Acids Res.

[66] Juncker AS, Willenbrock H, Von Heijne G, Brunak S, Nielsen H (2003). Prediction of lipoprotein signal peptides in Gram-negative bacteria.. Protein Sci.

[67] Ramos CR, Abreu PA, Nascimento AL, Ho PL (2004). A high-copy T7 Escherichia coli expression vector for the production of recombinant proteins with a minimal N-terminal His-tagged fusion peptide.. Braz J Med Biol Res.

[68] Pathirana RD, O'Brien-Simpson NM, Veith PD, Riley PF, Reynolds EC (2006). Characterization of proteinase-adhesin complexes of Porphyromonas gingivalis.. Microbiology.

[69] Lin YP, Lee DW, McDonough SP, Nicholson LK, Sharma Y (2009). Repeated domains of leptospira immunoglobulin-like proteins interact with elastin and tropoelastin.. J Biol Chem.

[70] Viratyosin W, Ingsriswang S, Pacharawongsakda E, Palittapongarnpim P (2008). Genome-wide subcellular localization of putative outer membrane and extracellular proteins in Leptospira interrogans serovar Lai genome using bioinformatics approaches.. BMC Genomics.

[71] Altschul SF, Madden TL, Schaffer AA, Zhang J, Zhang Z (1997). Gapped BLAST and PSI-BLAST: a new generation of protein database search programs.. Nucleic Acids Res.

[72] Altschul SF, Gish W, Miller W, Myers EW, Lipman DJ (1990). Basic local alignment search tool.. J Mol Biol.

